# Expression analysis of the estrogen receptor target genes in renal cell carcinoma

**DOI:** 10.3892/mmr.2014.2766

**Published:** 2014-10-24

**Authors:** ZHIHONG LIU, YOU LU, ZONGHAI HE, LIBO CHEN, YIPING LU

**Affiliations:** 1 Department of Urology, West China Hospital, Sichuan University, Chengdu, Sichuan 610041, P.R. China; 2 Department of Pediatrics, West China Second University Hospital, Sichuan University, Chengdu, Sichuan 610041, P.R. China

**Keywords:** renal cell carcinoma, differentially expressed genes, hormone, target receptor

## Abstract

The aim of the present study was to investigate the differentially expressed genes (DEGs) and target genes of the estrogen receptor (ER) in renal cell carcinoma. The data (GSE12090) were downloaded from the gene expression omnibus database. Data underwent preprocessing using the affy package for Bioconductor software, then the DEGs were selected via the significance analysis of microarray algorithm within the siggenes package. Subsequently, the DEGs underwent functional and pathway enrichment analysis using Database for Annotation Visualization and Integrated Discovery software. Following data analysis, transcriptional regulatory networks between the DEGs and transcription factors were constructed. Finally, the ER target genes were subjected to gene ontology enrichment analysis. A total of 215 DEGs were identified between the chromophobe renal cell carcinoma samples and the oncocytoma samples, including 126 upregulated and 89 downregulated genes. Functional enrichment analysis indicated that 25% of the DEGs were significantly enriched in functions associated with the plasma membrane. Among those DEGs, 105 were regulated by the ER. Further regulatory network analysis indicated that the ER was mainly involved in the regulation of oncogenes and tumor suppressor genes, including protease serine 8, claudin 7 and Ras-related protein Rab-25. In the present study, the identified ER target genes were demonstrated to be closely associated with tumor development; this knowledge may improve the understanding of the ER regulatory mechanisms during tumor development and promote the discovery of predictive markers for renal cell carcinoma.

## Introduction

Kidney cancer is a common urological malignancy that accounted for almost 3% of adult malignancies in 2007 (1). Statistics for 2010 indicated that >90,000 mortalities are caused by kidney cancer annually (2). Renal cell carcinoma, one of the most common subtypes of kidney cancer, originates in the lining of the proximal renal tubule and represents ~80% of cases of kidney cancer (3). For the treatment of renal cell carcinoma, surgery is the most common therapy, followed by chemotherapy and radiotherapy (4). However, the outcomes of these treatments are not satisfactory with a high recurrence rate of 20–40% (5). The lack of biomarkers for early detection and follow-up may lead to late diagnosis and subsequently to poor prognosis. Hence, a clear understanding of the pathogenesis of renal cell carcinoma is required in order to develop predictive biomarkers and target therapies.

Several important genes that participate in tumor development have been identified. One-allele inactivation of the von Hippel-Lindau (VHL) gene was identified in >90% of cases of non-inherited renal cell carcinoma (6). The inactivation of the VHL gene led to the production of a defective VHL protein, which would ordinarily degrade hypoxia-inducible factor (HIF) (7). A build-up of HIF led to its translocation to the nucleus, where it promotes the transcription of various genes critical to tumor development (8). Inactivated SET domain, bifurcated 1 and lysine-specific demethylase C, which are involved in histone modification, has been identified by sequencing in a previous study (9). These genes modify the methylation state of the lysine residues of histone H3 and regulate chromatin structure. The SWItch/sucrose nonfermentable chromatin remodeling complex gene and protein polybromo-a have also been implicated in the development of renal cell carcinoma (10).

These renal cell carcinoma-associated genes mainly regulate the expression of transcription factors and therefore influence tumor development. The estrogen receptor (ER), a hormone-regulated transcription factor, has been widely studied, and previous studies have demonstrated ER-regulated cell division and differentiation in the ovary, breast and uterus (11). Deregulation of ER transcriptional activity may lead to an increase in proliferation and cancer onset (12). Novel technologies, including high-throughput sequencing and microarray, have enabled a better understanding of ER regulatory mechanisms (13), and chromatin immunoprecipitation sequencing has been used to demonstrate that the ER binding sites are heterogeneous in human breast cancer cell lines and tissues (14,15). The binding sites of the ER in the chromosome are accompanied by multi-transcription factors (ER-cooperation factors) (11). Several ER target genes that participate in the cell cycle and cell proliferation have been previously identified, including cyclin-dependent kinase 6, CCAAT/enhancer binding protein alpha, disabled homolog 2, mitogen-responsive phosphoprotein and Janus kinase 2 (16).

Although the mechanism of the ER in breast cancer has been widely studied, its regulatory mechanisms in renal cell carcinoma development have not been investigated. In the present study, ER-regulated DEGs were identified, and were subsequently subjected to functional enrichment analysis. Furthermore, the interaction network between the transcription factors and their target genes was analyzed. The identification and function analysis of ER-specific genes may aid in the discovery of biomarkers for early detection and follow-up of renal cell carcinoma.

## Materials and methods

### Gene expression profiles

Gene expression data GSE12090 (17) were downloaded from the gene expression omnibus database (http://www.ncbi.nlm.nih.gov/geo/). The data were obtained from a total of 18 samples; 9 chromophobe renal cell carcinoma and 9 oncocytoma samples.

### Data preprocessing

The gene expression profiles (CEL format) were converted into expression values using the affy package in Bioconductor (18). The probe signal was converted into the corresponding gene symbol based on the microarray platform GPL570 [HG-U133_Plus_2] (Human Genome U133 Plus Array, version 2.0, Affymetrix, Santa Clara, CA, USA) using Bioconductor. For the genes corresponding to multiple probe sets, the average expression levels were used.

### DEG screening

The DEGs were identified using the significance analysis of microarray method (19) within the siggenes package. The criteria for selection were Δ=2.3 and a false discovery rate (FDR)<0.004. The Database for Annotation Visualization and Integrated Discovery (DAVID) online tool was used to perform the functional and pathway enrichment of DEGs in the present study. DAVID has integrated statistical methods for P-value adjustment, and the Benjamini method was used to adjust the P-value.

### Functional and pathway enrichment of the DEGs

Functional and pathway enrichment analysis of the DEGs were carried out using Database for Annotation Visualization and Integrated Discovery (20) software, based on the gene ontology (GO)and Kyoto Encyclopedia of Genes and Genomes (KEGG) pathway databases. P<0.05 was considered to indicate a statistically significant difference.

### Transcription regulatory network construction

The regulatory network between DEGs and transcription factors was constructed based on the target genes predicted using the University of California, Santa Cruz (UCSC) genome browser database (21). The regulatory network of the ER and its target genes was also constructed. Analysis of the network was conducted using Cytoscape software (version 3.0.0) (22).

### Functional enrichment analysis of ER target genes

The ER target genes were identified, and the upregulated and downregulated genes were subjected to GO functional enrichment analysis using the GO Enrichment Analysis Software Toolkit (23). P<0.05 was considered to indicate a statistically significant difference.

## Results

### Data preprocessing

**Figure 1:**
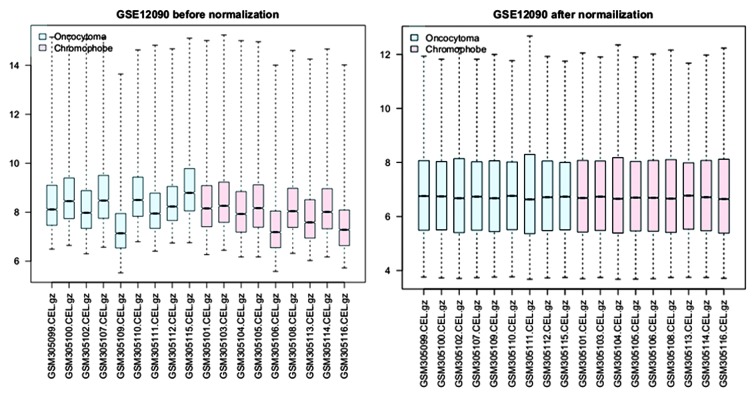
Gene expression data prior to normalization (left) and following normalization (right). Blue, oncocytoma; red, chromophobe renal cell carcinoma. Horizontal axis, sample; vertical axis, expression value. The black line in the colored box indicates the median expression value.

A total of 19,944 gene expression values were obtained from the 18 samples following data preprocessing. The normalized gene expression data were compared with the raw data in subsequent analysis (Fig. 1). The median expression values were nearly the same following normalization.

### DEG screening

**Figure 2:**
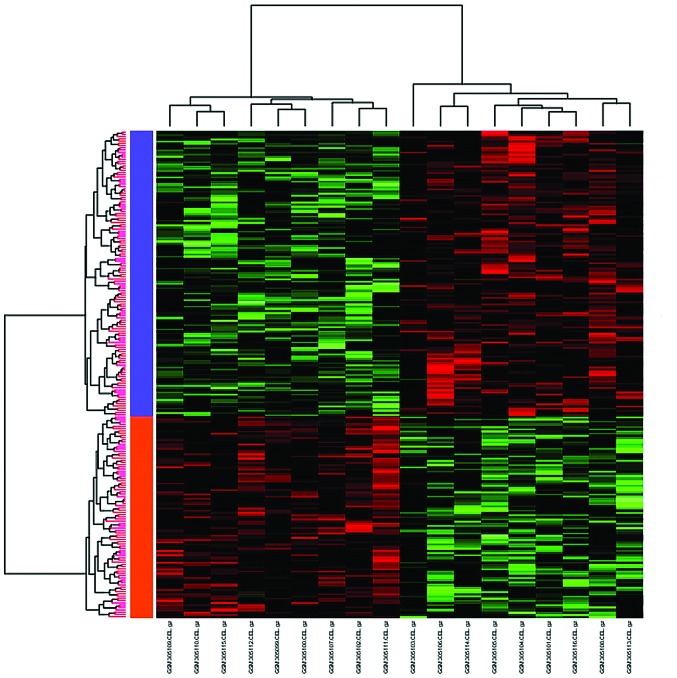
Clustering analysis of DEGs. A darker red in the heat-map indicates a stronger upregulation in expression and a darker green indicates a stronger downregulation in expression. The horizontal X-axis lists the samples being clustered; on the left vertical colored bar, the orange indicates the upregulated DEGs and the purple indicates the downregulated DEGs. DEG, differentially expressed genes.

A total of 215 DEGs were identified with the abovementioned criteria (Δ=2.3 and FDR<0.004) between the chromophobe renal cell carcinoma and oncocytoma samples. Among those DEGs, 126 genes were upregulated and 89 genes were downregulated (Table I). The DEGs were then subjected to clustering analysis: The samples were clustered into two groups, namely the upregulation group and the downregulation group (Fig. 2). The upregulated genes were labeled in orange, while the downregulated genes were labeled in purple.

### Functional and pathway enrichment analysis of DEGs

The top 10 GO terms of the upregulated and downregulated DEGs are presented in Table II. Nearly one quarter of the DEGs were associated with the plasma membrane. Pathway enrichment analysis indicated that the upregulated genes were enriched in two KEGG pathways: Cell conjunction and phosphatidylinositol signaling conduction. However, the downregulated genes were not significantly enriched in any KEGG pathway.

### Transcriptional regulatory network construction

**Figure 3:**
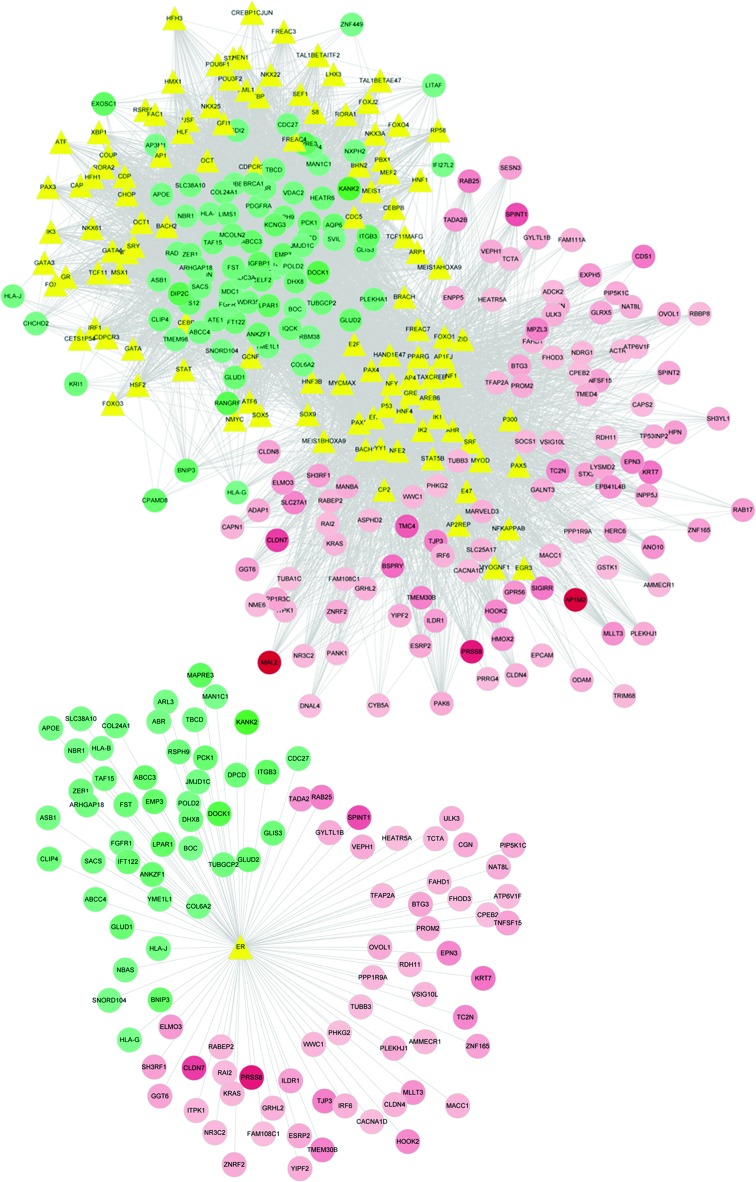
Transcriptional regulatory network of DEGs (left) and the regulatory network between the ER and its target genes (right). Yellow, transcription factor; red, upregulated genes; and green, downregulated genes. DEG, differentially expressed gene; ER, estrogen receptor.

The transcription factors that regulate DEG expression were predicted using the UCSC database. A total of 115 transcription factors were identified, and the interaction network between the transcription factors and DEGs was constructed (Fig. 3). Further analysis indicated that the ER participated in the regulation of 105 DEGs, of which 59 were upregulated and 46 were downregulated. Based on analysis of the regulatory network of the ER and DEGs, it was possible to deduce that the ER is involved in the regulation of oncogene and tumor suppressor gene expression (Fig. 3).

### GO functional enrichment analysis of ER target genes

The DEGs regulated by the ER were subjected to GO functional enrichment analysis (Table III). The downregulated genes were demonstrated to be involved in oxidoreductase activity, leucine binding and glutamate dehydrogenase-NAD(P)^+^ activity; while the upregulated genes were associated with occluded and tight junctions as well as apical junction complexes.

## Discussion

In the present study, 215 DEGs were identified, of which 126 were upregulated and 89 downregulated. Functional enrichment analysis indicated that 25% of the DEGs were significantly enriched in functions associated with the plasma membrane. Among those DEGs, 105 were possibly regulated by the ER. Following regulatory network analysis, it was demonstrated that the ER mainly regulated the expression of oncogenes and tumor suppressor genes. The DEGs that were regulated by the ER were then subjected to systematic analysis.

Several DEGs have been demonstrated to be associated with tumor development, including protease serine 8 (*PRSS8*), claudin 7 (*CLDN7*) and Ras-related protein Rab-25 (*RAB25*). These three genes were most significantly upregulated in renal cell carcinoma and may be important in tumor development.

PRSS8 encodes a trypsinogen protein that belongs to the trypsin family of serine proteases. Serine proteases are involved in the regulation of snail family zinc finger 2 and E-cadherin expression in cancer cells (24,25). Additionally, the differential expression of *PRSS8* has been identified in prostate, breast, gastric and ovarian cancer cases (26), and the downregulation of *PRSS8* in these cases of epithelial cancer was attributed to DNA hypermethylation (27,28). Hence, the upregulation of *PRSS8* by the ER is likely to have enhanced DNA hypermethylation and led to the regulation of the expression of genes associated with renal cell carcinoma.

CLDN7 is an integral membrane protein that has been observed to be differentially expressed in ovarian and esophageal squamous cell carcinoma cells (29,30). In a previous study, CLDN7 was demonstrated to be significantly differentially expressed in ovarian carcinoma, based on CLDN7 expression analysis at the mRNA and protein levels in 110 patients with epithelial ovarian carcinoma (31). In esophageal squamous cell carcinoma cells, CLDN7 is often absent or localized to the cytoplasm, rather than confined to the cell membrane as in normal esophageal cells (32) In addition, the dysregulation of CLDN7 may lead to decreased E-cadherin expression, loss of epithelial architecture and an increase in the invasion observed in squamous cell carcinoma. This evidence indicates that CLDN7 may promote tumor development by disrupting the cell adhesion process.

RAB25 belongs to the RAS superfamily and serves a crucial function in vesicle trafficking, signal transduction and receptor recycling (33). RAB25 has been observed to be upregulated in prostate and ovarian cancer, and is correlated with poor prognosis (34). However, up- and downregulation of RAB25 has been documented in breast cancer (35). The overexpression of RAB25 may promote cellular bioenergetics and hence inhibit apoptosis and autophagy (36). Another study suggested that RAB25, when combined with the chloride intracellular channel 3, regulates tumor invasiveness and mediates the recycling of α5β1-integrin to the plasma membrane from a late endosomal compartment (37). This evidence indicates that RAB25 is crucial in determining tumor development, progression and aggressiveness (38). Therefore, the upregulation of RAB25 in renal cell carcinoma may promote tumor development.

The DEG function analysis conducted in the present study indicated that the regulatory mechanism of ER in renal cell carcinoma is complex. The functional enrichment analysis demonstrated that the ER target genes mainly regulated transmembrane receptor and protein tyrosine kinase activity, which may serve a pivotal role in multiple diseases. The transmembrane G protein-coupled receptors are widely used as drug targets for various diseases, and particularly for cancer (39). The ER participates in the regulation of protein tyrosine kinase activity, which is an important signaling pathway in cell proliferation. The dysregulation of tyrosine kinases has verified its association with breast cancer and diverse biological functions (40). Sun *et al* (41) observed that multiple proto-oncogenic tyrosine kinases were activated by loss of the PTPN12 (protein tyrosine phosphatase non-receptor type 12) phosphatase in breast cancer. Therefore, the regulation of ER target genes may significantly influence the development of renal cell carcinoma.

In conclusion, the DEGs regulated by the ER in renal cell carcinoma were identified and analyzed in the present study. The interaction network and functional enrichment analysis demonstrated that the ER regulates the expression of oncogenes and tumor suppressor genes. Therefore, the present study enhanced the understanding of the mechanism of the regulation of the ER during tumor development and may aid in the discovery of predictive markers for renal cell carcinoma.

## Figures and Tables

**Table I:** The top 10 of up- and downregulated DEGs.

Number	Gene	D-value
Upregulated		
1	*ESRP1*	24.25
2	*MAL2*	19.79
3	*AP1M2*	18.69
4	*PRSS8*	15.42
5	*CLDN7*	12.53
6	*SPINT1*	11.46
7	*TMC4*	10.71
8	*BSPRY*	−10.61
9	*KRT7*	9.56
10	*CDS1*	9.12
Downregulated		
1	*NBL1*	−11.01
2	*KANK2*	−10.61
3	*DOCK1*	−8.72
4	*DIP2C*	−8.35
5	*RANGRF*	−8.16
6	*MAPRE3*	−7.63
7	*IGFBP1*	−7.34
8	*EXOSC1*	−7.32
9	*ITGB3*	−7.26
10	*LPAR1*	−7.08

All Q-values = 0. DEG, differentially expressed gene.

**Table II:** GO and KEGG pathway enrichment results.

A, Upregulated DEGs			

Category	Term and function	Count (n)	P-value
GOTERM_CC_FAT	GO:0005923~tight junction	5	0.00
GOTERM_CC_FAT	GO:0070160~occluding junction	5	0.00
GOTERM_CC_FAT	GO:0009898~internal side of plasma membrane	8	0.00
GOTERM_CC_FAT	GO:0043296~apical junction complex	5	0.00
GOTERM_CC_FAT	GO:0016327~apicolateral plasma membrane	5	0.00
GOTERM_CC_FAT	GO:0005911~cell-cell junction	6	0.01
GOTERM_CC_FAT	GO:0044459~plasma membrane part	23	0.01
GOTERM_CC_FAT	GO:0005886~plasma membrane	34	0.01
GOTERM_CC_FAT	GO:0000267~cell fraction	14	0.01
GOTERM_CC_FAT	GO:0005626~insoluble fraction	11	0.03
KEGG_PATHWAY	hsa04530:Tight junction	6	0.00

B, Downregulated DEGs			

Category	Term	Count (n)	P-value

GOTERM_CC_FAT	GO:0015630~microtubule cytoskeleton	9	0.00
GOTERM_CC_FAT	GO:0042612~MHC class I protein complex	3	0.01
GOTERM_MF_FAT	GO:0070728~leucine binding	2	0.01
GOTERM_MF_FAT	GO:0004353~glutamate dehydrogenase [NAD(P)^+^] activity	2	0.01
GOTERM_MF_FAT	GO:0000166~nucleotide binding	18	0.02
GOTERM_BP_FAT	GO:0045137~development of primary sexual characteristics	4	0.02
GOTERM_BP_FAT	GO:0000077~DNA damage checkpoint	3	0.02
GOTERM_BP_FAT	GO:0031570~DNA integrity checkpoint	3	0.02
GOTERM_BP_FAT	GO:0006974~response to DNA damage stimulus	6	0.03
GOTERM_CC_FAT	GO:0044430~cytoskeletal part	10	0.03

GO, gene ontology; KEGG, Kyoto Encyclopedia of Genes and Genomes; DEG, differentially expressed gene. P<0.05 was considered to indicate a statistically significant difference; hsa, *Homo sapiens*; MHC, major histocompatibility complex.

**Table III:** GO enrichment analysis of the ER target genes.

A, Downregulated ER target genes			

Category	Term	Count (n)	P-value
GOTERM_CC_FAT	GO:0042612~MHC class I protein complex	3	0.00207
GOTERM_MF_FAT	GO:0016639~oxidoreductase activity, acting on the CH-NH_2_ group of donors, NAD or NADP as acceptor	2	0.00477
GOTERM_MF_FAT	GO:0070728~leucine binding	2	0.00477
GOTERM_MF_FAT	GO:0004353~glutamate dehydrogenase [NAD(P)^+^] activity	2	0.00477
GOTERM_MF_FAT	GO:0004352~glutamate dehydrogenase activity	2	0.00477
GOTERM_CC_FAT	GO:0042611~MHC protein complex	3	0.00836
GOTERM_CC_FAT	GO:0015630~microtubule cytoskeleton	6	0.00966
GOTERM_MF_FAT	GO:0001883~purine nucleoside binding	10	0.01032
GOTERM_MF_FAT	GO:0001882~nucleoside binding	10	0.01078
GOTERM_BP_FAT	GO:0006538~glutamate catabolic process	2	0.01455

B, Upregulated ER target genes			

Category	Term	Count (n)	P-value

GOTERM_CC_FAT	GO:0070160~occluding junction	4	0.00086
GOTERM_CC_FAT	GO:0005923~tight junction	4	0.00086
GOTERM_CC_FAT	GO:0043296~apical junction complex	4	0.00208
GOTERM_CC_FAT	GO:0016327~apicolateral plasma membrane	4	0.00226
GOTERM_CC_FAT	GO:0044459~plasma membrane part	13	0.00668
GOTERM_CC_FAT	GO:0009898~internal side of plasma membrane	5	0.00852
GOTERM_CC_FAT	GO:0030054~cell junction	6	0.00995
GOTERM_CC_FAT	GO:0005911~cell-cell junction	4	0.01271
GOTERM_BP_FAT	GO:0010324~membrane invagination	4	0.02042
GOTERM_BP_FAT	GO:0006897~endocytosis	4	0.02042

P<0.05 was considered to indicate a statistically significant difference. GO, gene ontology; ER, estrogen receptor; MHC, major histocompatibility complex.
